# Genome-wide identification and expression analysis of the bZIP transcription factors, and functional analysis in response to drought and cold stresses in pear (*Pyrus breschneideri*)

**DOI:** 10.1186/s12870-021-03356-0

**Published:** 2021-12-09

**Authors:** Ming Ma, Qiming Chen, Huizhen Dong, Shaoling Zhang, Xiaosan Huang

**Affiliations:** State Key Laboratory of Crop Genetics and Germplasm Enhancement, Centre of Pear Engineering Technology Research, Nanjing Agricultural University, Nanjing, China

**Keywords:** bZIP transcription factor family, Chinese white pears, Drought and cold stress tolerance, Evolutionary pattern, Gene expression

## Abstract

**Background:**

Transcription factors (TFs) are involved in many important biological processes, including cell stretching, histological differentiation, metabolic activity, seed storage, gene regulation, and response to abiotic and biotic stresses. Little is known about the functions, evolutionary history, and expression patterns of basic region-leucine zipper TF family genes in pear, despite the release of the genome of Chinese white pears (“Dangshansuli”).

**Results:**

Overall, 92 bZIP genes were identified in the pear genome (*Pyrus breschneideri*). Of these, 83 were randomly distributed on all 17 chromosomes except chromosome 4, and the other 9 genes were located on loose scaffolding. The genes were divided into 14 subgroups. Whole-genome duplications, dispersed duplication, and purifying selection for whole-genome duplications are the main reasons for the expansion of the *PbrbZIP* gene family. The analysis of functional annotation enrichment indicated that most of the functions of *PbrbZIP* genes were enriched in Gene Ontology and Kyoto Encyclopedia of Genes and Genomes pathways involved in the abiotic stress response. Next, expression analysis and virus-induced gene silencing results indicated that *PbrbZIP* genes might play critical roles in response to drought and cold stresses, especially for the genes from subgroups A, C, G, I, and S.

**Conclusions:**

Ninety-two *PbrbZIP* genes were identified from the pear genome and classified into 14 subgroups. *PbrbZIP* genes were mainly expanded from whole-genome duplications and dispersed duplications and retained by purifying selection. *PbrbZIP* genes were induced by cold and drought stresses and played important roles in drought and cold tolerance. These results provided useful information for further increasing the tolerance of pears to stresses and a foundation to study the cold and drought tolerance mechanism of *PbrbZIP* genes.

**Supplementary Information:**

The online version contains supplementary material available at 10.1186/s12870-021-03356-0.

## Background

Transcription factors (TFs) play essential regulatory roles in many crucial biological processes in plants. Knowing the functional properties of TFs by understanding the biological processes in which they are involved is necessary. Up to now, about 64 TF families have been reported in plants [1]. The basic family of leucine zippers (bZIP) is one of the largest and most diverse families [1, 2]. They are characterized by a conserved bZIP domain of 40–80 amino acids, with 2 structural features. A basic region of DNA binding (N-X7-R/K-X9) was used for sequence-specific DNA binding, and a several-heptad repeat sequence consisting of leucine or other hydrophobic amino acids (such as Ile, Val, Phe, or Met) made up the bZIP motif for dimeric specificity [2–4].

bZIP TFs are involved in several important biological processes, such as cell stretching [5, 6], histological differentiation [7, 8], metabolic activity [9], seed storage protein gene regulation, and embryogenesis and seed maturation [10]. bZIP TFs take part in responding to abiotic and biotic stresses, including hormone and sugar signaling [11, 12], photoreaction [13, 14], pathogen defense [15, 16], and abiotic stresses tolerance [17, 18]. According to existing studies, bZIP TF plays an important role in plant response to abiotic stresses, such as drought, cold, salt, abscisic acid (ABA), and mechanical damage [19, 20]. In soybeans, *GmbZIP44*, *GmbZIP62*, or *GmbZIP78* TFs may enhance salt and cold tolerance [21]. *OsbZIP62* intervenes in the signaling pathways of ABA and regulates positively the drought tolerance of rice by regulating the expression of genes associated with stress [22]. *ZmbZIP4* TF can enhance the ability of corn to resist abiotic stresses by regulating ABA synthesis and root development [23]. In grapes, *VlbZIP36* improves drought tolerance due to the transcriptional regulation of ABA/stress-related genes [24]. *MdHY5* positively modulates the cold tolerance in apple calli [25].

To date, the bZIP TF families were identified or predicted across multiple plant genomes. A total of 75 *bZIP* genes were first found in *Arabidopsis thaliana* [3]. Wolfgang Drföge-Laser and co-workers classified the 78 bZIP members of *A. thaliana* into 13 subgroups [26], 89 in rice (*Oryza sativa*) [2], 131 in soybean (*Glycine max*) [21], 125 in maize (*Zea mays*) [27], 55 in the grapevine (*Vitis vinifera*) genome [28], and 112 *bZIP* genes in apple (*Malus domestica* Borkh) [29]. No studies have reported on the bZIP family in pears despite pears being an important cash crop widespread worldwide.

In fact, abiotic stresses, such as low temperature and drought, not only limit the cultivation area but also affect the growth and yield of pears. This situation needs to be addressed urgently. *PbrBAM3* increases the cold tolerance of pears by increasing the antioxidant activity and soluble sugar levels [30]. *PbrWRKY53* positively regulates ascorbic acid (AsA) biosynthetic activity and enhances the drought tolerance of pears by regulating AsA-mediated reactive oxygen species (ROS) scavenging [31]. Recent advances in genomics and gene technology provide many new molecular tools for improving crop resistance to biological stresses [32]. The genome sequence of *Pyrus bretschneideri* was released in 2013 [33], providing an opportunity for genome-level identification, analysis of protein families, and genetic improvement using candidate genes for stress resistance.

In this study, 92 *PbrbZIP* genes were identified from the Chinese white pear genome. Sequence and phylogenetic analyses were performed to determine the relationships among these genes. The results of the analysis of protein profiles and intron/exon structures supported the classification of the *PbrbZIP* family. Whole-genome duplications (WGD)/segmental and dispersed duplications probably led to the expansion of the *bZIP* family. In addition, RNA-seq data showed that *PbrbZIP* genes had different expression patterns under drought and cold stresses. The results of this study might help better understand the role of bZIP TF in the abiotic stresses response of pears and provide a foundation for identifying candidate genes involved in the cold and drought tolerance of pears.

## Results

### Identification of bZIP TFs in Chinese white pears

Local Hidden Markov Model (HMM) files (PF00170, PF07716, and PF07777) were used to identify the *bZIP* gene in the Chinese white pear genome. A total of 96 candidate PbrbZIP protein sequences were identified. The Simple Modular Architecture Research Tool (SMART) (http://smart.embl-heidelberg.de/) and the National Center for Biotechnology Information (NCBI) Batch CD-Search tools were used to check for the presence of bZIP conserved domains, and redundant sequences were removed. A total of 92 putative *bZIP* genes were identified; the nomenclature and associated information are listed in Table 1 and Table S1. These *PbrbZIP* genes were named through *PbrbZIP01* to *PbrbZIP92* based on the order of the gene ID. A total of 83 *PbrbZIP* genes were randomly distributed on all 17 chromosomes except chromosome 4, and the other 9 genes were located on loose scaffoldings. Chromosome 15 had the most *PbrbZIP* genes (11 genes), and chromosome 16 had only one gene. Protein molecular weights of *PbrbZIP* genes ranged from 14.03 to 79.84 KDa. Protein isoelectric points ranged from 5.04 to 10.51, with 54 below 7 (Table 1). The PbrbZIP proteins might be soluble because of their positive grand average of hydropathy, which was consistent with its potential function as TF.

**Table 1:** Characteristics of identified PbrbZIP proteins

Name	ID	Chr.No	ORF	Strat	End	Extron num	MW(KDA)	PI	GRAVY
*PbrbZIP01*	*Pbr001076.1*	Chr2	702	12231889	1.2E+ 07	1	26.49	10.41	− 0.685
*PbrbZIP02*	*Pbr002338.1*	scaffold1099.0	1260	54879	61696	12	43.16	6.62	−0.801
*PbrbZIP03*	*Pbr002470.1*	Chr17	618	16705451	1.7E+ 07	4	23.73	8.95	−0.618
*PbrbZIP04*	*Pbr002622.1*	Chr15	510	999456	1002190	4	18.34	9.34	−0.931
*PbrbZIP05*	*Pbr002928.1*	Chr7	726	12429663	1.2E+ 07	4	27.24	9.51	−0.907
*PbrbZIP06*	*Pbr002981.1*	Chr7	726	12802639	1.3E+ 07	4	27.24	9.51	−0.907
*PbrbZIP07*	*Pbr003516.1*	Chr3	1263	16858552	1.7E+ 07	4	46.99	6.58	−0.875
*PbrbZIP08*	*Pbr003518.1*	Chr3	1542	16831076	1.7E+ 07	12	56.42	6.03	−0.548
*PbrbZIP09*	*Pbr003750.1*	scaffold1170.0	726	35506	37807	4	27.18	9.58	−0.876
*PbrbZIP10*	*Pbr004364.1*	Chr12	1461	2057108	2060551	6	53.27	6.21	−0.851
*PbrbZIP11*	*Pbr005556.1*	scaffold1282.0	1584	9370	12132	4	57.59	6.79	−0.912
*PbrbZIP12*	*Pbr005557.1*	scaffold1282.0	1734	15287	18296	4	62.81	6.63	−0.91
*PbrbZIP13*	*Pbr005860.1*	Chr15	1734	2664599	2667542	4	62.88	6.66	−0.901
*PbrbZIP14*	*Pbr005861.1*	Chr15	1584	2671458	2674216	4	57.57	6.79	−0.918
*PbrbZIP15*	*Pbr005914.1*	Chr15	474	3000081	3001635	1	17.69	7.07	−0.731
*PbrbZIP16*	*Pbr006046.1*	scaffold1301.0	669	48	1811	4	24.19	9.22	−0.623
*PbrbZIP17*	*Pbr007163.1*	Chr14	639	14914116	1.5E+ 07	1	22.57	7.84	−0.661
*PbrbZIP18*	*Pbr007566.2*	Chr14	1740	135984	139566	6	62.38	7.59	−0.746
*PbrbZIP19*	*Pbr007589.1*	Chr14	1326	344099	346885	4	48.15	8.28	−0.616
*PbrbZIP20*	*Pbr008557.1*	Chr8	1086	2230491	2232813	8	40.94	6.81	−0.528
*PbrbZIP21*	*Pbr008558.1*	Chr8	987	2197741	2199160	3	36.48	9.32	−0.629
*PbrbZIP22*	*Pbr009074.1*	Chr10	1047	10127839	1E+ 07	6	35.36	9.97	−0.842
*PbrbZIP23*	*Pbr009262.1*	Chr15	1047	3989301	3992176	6	37.95	5.37	−0.591
*PbrbZIP24*	*Pbr009654.1*	Chr7	2229	1362620	1366901	2	79.84	6.02	−0.558
*PbrbZIP25*	*Pbr009693.1*	Chr7	510	1703894	1704403	1	18.99	6.23	−0.876
*PbrbZIP26*	*Pbr010436.1*	scaffold170.2.1	969	93882	98146	3	35.94	5.94	−0.842
*PbrbZIP27*	*Pbr010517.1*	Chr5	1047	2957830	2961646	4	38.24	6.71	−0.936
*PbrbZIP28*	*Pbr012802.1*	Chr2	1278	5526442	5528863	6	47.85	6.9	−0.658
*PbrbZIP29*	*Pbr013043.1*	Chr3	1131	22992061	2.3E+ 07	5	41.34	8.92	−0.892
*PbrbZIP30*	*Pbr013133.1*	Chr3	1317	22249661	2.2E+ 07	4	46.86	6.11	−0.799
*PbrbZIP31*	*Pbr013209.1*	Chr3	615	21719032	2.2E+ 07	1	23.48	6.3	−0.841
*PbrbZIP32*	*Pbr013267.1*	Chr3	1362	21256215	2.1E+ 07	9	50.43	6.1	−0.399
*PbrbZIP33*	*Pbr014120.1*	Chr6	957	9323825	9326350	6	32.7	5.78	−0.679
*PbrbZIP34*	*Pbr014592.1*	Chr5	1350	22847185	2.3E+ 07	5	49.69	6.3	−0.794
*PbrbZIP35*	*Pbr014594.1*	Chr5	1533	22872574	2.3E+ 07	12	56.13	6.03	−0.531
*PbrbZIP36*	*Pbr015119.3*	Chr6	1002	19889864	2E+ 07	2	37.23	5.48	−0.456
*PbrbZIP37*	*Pbr015675.1*	Chr2	1002	6170887	6178380	8	37.25	8.59	−0.615
*PbrbZIP38*	*Pbr016302.1*	Chr6	1284	21111673	2.1E+ 07	12	43.54	6.88	−0.887
*PbrbZIP39*	*Pbr016568.1*	Chr17	609	17890291	1.8E+ 07	1	23.37	5.9	−0.987
*PbrbZIP40*	*Pbr017262.1*	Chr15	1050	20035048	2E+ 07	11	36.44	5.53	−0.906
*PbrbZIP41*	*Pbr017284.1*	Chr11	1050	24819436	2.5E+ 07	5	38.31	9.49	−0.798
*PbrbZIP42*	*Pbr017778.1*	Chr12	1320	20360616	2E+ 07	4	47.73	8.76	−0.641
*PbrbZIP43*	*Pbr017979.1*	Chr17	459	19768805	2E+ 07	1	17.64	9.65	−0.756
*PbrbZIP44*	*Pbr018534.1*	Chr13	480	7268883	7270283	1	17.92	7.11	−0.732
*PbrbZIP45*	*Pbr018536.1*	Chr13	429	7319005	7319433	1	15.63	9.45	−0.78
*PbrbZIP46*	*Pbr018746.1*	Chr8	945	10553837	1.1E+ 07	6	34.2	5.04	−0.473
*PbrbZIP47*	*Pbr019461.1*	Chr10	921	22800599	2.3E+ 07	5	33.89	8.86	−0.759
*PbrbZIP48*	*Pbr019779.1*	Chr15	459	6992833	6993291	1	17.77	6.97	−0.753
*PbrbZIP49*	*Pbr020210.1*	Chr6	465	4248339	4248803	1	17.7	8.04	−0.818
*PbrbZIP50*	*Pbr020743.1*	Chr10	807	17290780	1.7E+ 07	2	29.31	6.15	−0.686
*PbrbZIP51*	*Pbr021041.1*	Chr1	1272	3250368	3253437	12	45.17	8.69	−0.697
*PbrbZIP52*	*Pbr022222.1*	Chr9	609	18881465	1.9E+ 07	1	23.28	5.74	−0.885
*PbrbZIP53*	*Pbr022503.1*	Chr17	888	2569875	2573351	4	32.79	6.21	−0.726
*PbrbZIP54*	*Pbr022685.1*	Chr3	429	1149894	1151224	1	16.31	9.09	−0.806
*PbrbZIP55*	*Pbr022894.1*	Chr2	2187	7309361	7312996	2	78.57	6.85	−0.511
*PbrbZIP56*	*Pbr022933.1*	Chr2	738	6976334	6977338	1	27.18	7.12	−0.783
*PbrbZIP57*	*Pbr023279.1*	Chr2	675	15827845	1.6E+ 07	1	23.86	9.64	−0.626
*PbrbZIP58*	*Pbr024746.1*	Chr2	909	8549572	8555592	2	33.82	5.67	−0.775
*PbrbZIP59*	*Pbr025283.1*	Chr5	1377	19163038	1.9E+ 07	6	49.73	6.03	−0.676
*PbrbZIP60*	*Pbr026554.1*	Chr8	1143	4008765	4014636	4	42.93	7.24	−0.982
*PbrbZIP61*	*Pbr026723.2*	Chr14	918	8763592	8766217	7	33.09	5.09	−0.708
*PbrbZIP62*	*Pbr026741.1*	Chr3	1062	2967110	2969439	4	39.75	6.22	−0.976
*PbrbZIP63*	*Pbr026913.1*	Chr15	1089	28456413	2.8E+ 07	8	41.09	6.33	−0.532
*PbrbZIP64*	*Pbr027414.1*	Chr5	774	12967287	1.3E+ 07	1	27.88	5.92	−0.621
*PbrbZIP65*	*Pbr027468.1*	Chr13	1572	2302476	2305727	10	58.54	5.98	−0.471
*PbrbZIP66*	*Pbr027818.1*	Chr15	495	9823393	9826153	4	17.78	9	−0.984
*PbrbZIP67*	*Pbr028080.1*	Chr8	1728	14864215	1.5E+ 07	4	62.9	6.92	−0.931
*PbrbZIP68*	*Pbr028081.1*	Chr8	1515	14868512	1.5E+ 07	4	54.73	5.85	−0.842
*PbrbZIP69*	*Pbr028249.1*	Chr12	486	5166957	5168690	4	18.19	9.12	−0.814
*PbrbZIP70*	*Pbr028659.1*	Chr12	1359	12490432	1.2E+ 07	11	50.2	8.83	−0.646
*PbrbZIP71*	*Pbr029239.1*	Chr1	459	2389878	2390653	1	17.64	9.65	−0.756
*PbrbZIP72*	*Pbr029701.1*	Chr9	1044	14021920	1.4E+ 07	11	36.27	5.74	−0.887
*PbrbZIP73*	*Pbr030038.1*	Chr13	615	4056908	4057801	1	23.47	5.93	−0.751
*PbrbZIP74*	*Pbr030476.1*	Chr5	861	2296058	2297044	2	30.98	5.12	−0.585
*PbrbZIP75*	*Pbr030604.1*	Chr9	1341	19283974	1.9E+ 07	10	49.78	7.28	−0.65
*PbrbZIP76*	*Pbr030829.1*	Chr11	426	565147	566497	1	16.24	6.75	−0.791
*PbrbZIP77*	*Pbr031203.1*	Chr15	1053	39848596	4E+ 07	4	36.78	6.61	−0.789
*PbrbZIP78*	*Pbr033760.1*	Chr15	603	30168150	3E+ 07	1	22.97	10.51	−0.659
*PbrbZIP79*	*Pbr034805.1*	Chr13	846	14373472	1.4E+ 07	3	30.55	9.65	−0.723
*PbrbZIP80*	*Pbr035554.1*	Chr5	369	15980857	1.6E+ 07	3	14.03	9.38	−0.475
*PbrbZIP81*	*Pbr035863.1*	Chr12	1338	16253048	1.6E+ 07	10	49.71	6.98	−0.642
*PbrbZIP82*	*Pbr036339.1*	Chr10	1026	19091541	1.9E+ 07	4	37.35	6.6	−0.825
*PbrbZIP83*	*Pbr036605.1*	Chr11	1539	18152763	1.8E+ 07	11	56.54	6.75	−0.53
*PbrbZIP84*	*Pbr037165.1*	Chr16	1230	19058863	1.9E+ 07	12	43.64	6.62	−0.769
*PbrbZIP85*	*Pbr038249.1*	Chr11	1056	4628100	4630519	4	39.69	6.27	−0.977
*PbrbZIP86*	*Pbr039911.1*	scaffold868.0	1134	46463	50532	4	42.23	7.2	−0.893
*PbrbZIP87*	*Pbr039916.1*	scaffold868.0	1134	103303	107372	4	42.23	7.2	−0.893
*PbrbZIP88*	*Pbr040390.1*	scaffold888.0	1368	59641	62060	4	49.53	9.43	−0.585
*PbrbZIP89*	*Pbr040479.1*	Chr2	474	15517927	1.6E+ 07	1	17.9	5.41	−0.794
*PbrbZIP90*	*Pbr041663.1*	Chr7	1002	2182780	2189697	8	17.9	5.41	−0.794
*PbrbZIP91*	*Pbr042765.1*	Chr10	807	17608035	1.8E+ 07	2	29.34	6.15	−0.695
*PbrbZIP92*	*Pbr042848.1*	Chr8	780	6290536	6291676	1	27.28	5.85	−0.673

### Phylogenetic analysis and classification of the pear *bZIP* gene family

**Fig. 1:**
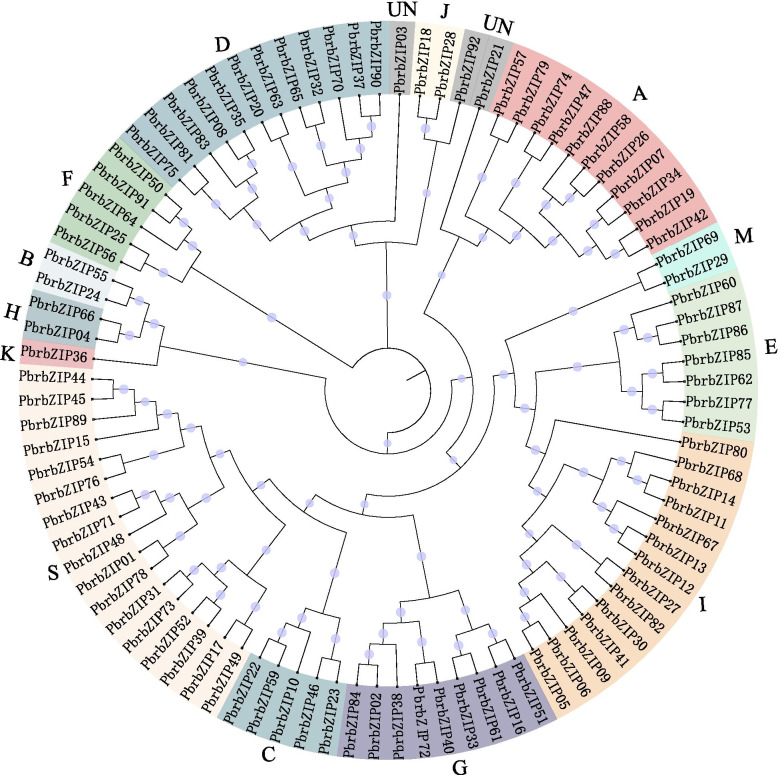
Un-rooted phylogenetic tree of PbrbZIP proteins. MEGA 7 was used to construct the phylogenetic tree based on the protein sequences. iTOL (https://itol.embl.de/) was used to annotate and review the phylogenic tree. The proteins were clustered into 14 groups. Different background colors indicate the different group of the PbrbZIP proteins

**Fig. 2:**
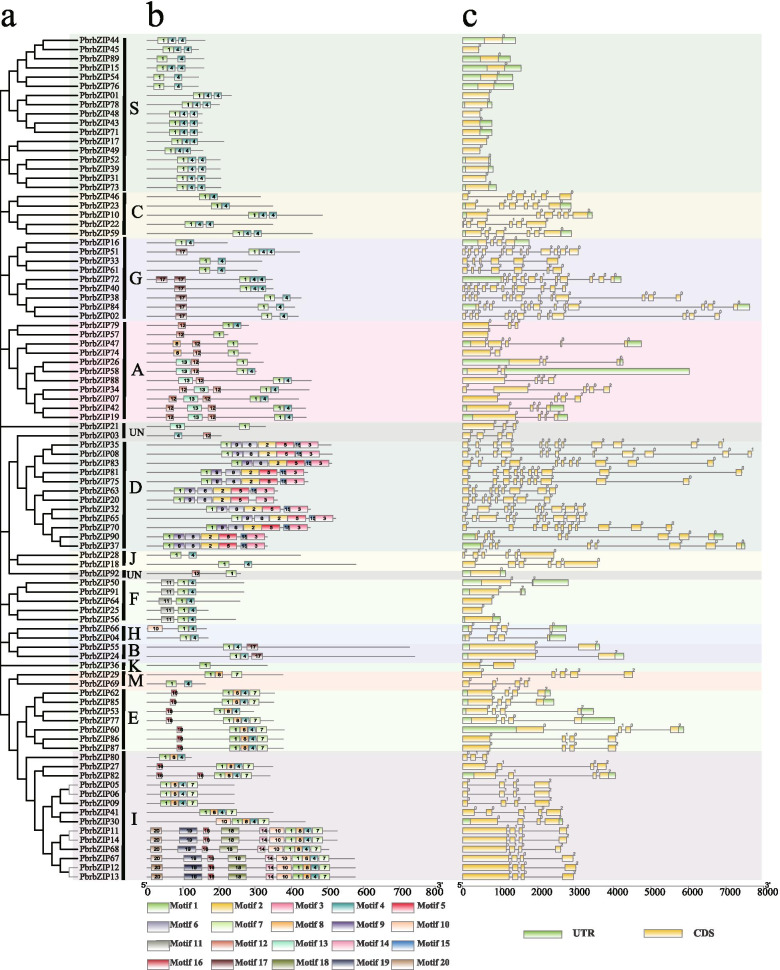
Gene structure schematics and preserved motifs patterns in the *PbrbZIP* family. **a** Subgroup classification: The phylogenetic tree was derived from 92 PbrbZIP genes with MEGA 7. **b** Conserved motif analysis: 20 separate patterns were identified with the MEME suite and each pattern was depicted with different colors. **c** Gene structural analysis

An unrooted neighbor-joining phylogenetic tree was built to classify these genes, and the evolutionary relationship of the *PbrbZIP* gene was studied (Figs. 1, 2a, and S1). *PbrbZIP* genes were divided into 14 subgroups (A, B, C, D, E, F, G, H, I, J, K, M, S, and UN) based on the relationship with *A. thaliana bZIP* genes. *PbrbZIP03*, *PbrbZIP21*, and *PbrbZIP92* (subgroup UN; Fig. 1) formed three small, unique subgroups in the phylogenetic tree and might have evolutionary trajectories unrelated to other subgroups. Subgroup S had the largest number of *PbrbZIP* genes (17 genes), followed by subgroups I (14 genes) and A (11 genes). Subgroup K had only one gene *(PbrbZIP36*).

The potential function of these genes could be depicted based on the annotation information of Gene Ontology (GO) and Kyoto Encyclopedia of Genes and Genomes (KEGG) databases. Functional enrichment analysis was performed to forecast the potential functions of *PbrbZIP* genes. *PbrbZIP* genes were enriched mainly in transcription regulator activity, molecular function, DNA-binding TF activity, biosynthetic process, and some regulatory functions; the regulation of the expression of TFs was closely related to all of these functions (Fig. S2a). In addition, the KEGG enrichment result showed that these genes were enriched only in plant hormone signal transduction, circadian rhythm, and mitogen-activated protein kinase (MAPK) signaling, and these mechanisms were related mainly to the regulation of downstream gene expression by bZIP family TFs (Fig. S2b). The crucial TFs of these pathways were also discovered through BLASTP. *ATbZIP56* (*HY5*), whose orthologous genes were *PbrbZIP66* and *PbrbZIP04*, integrated hormonal signaling pathways (auxin, gibberellin, brassinolide, and ethylene) and interacted with the promoter of the monoterpene synthase gene *QH6* in modulating its rhythmic expression [34, 35]. *ATbZIP51* (the ortholog of *PbrbZIP82* and *PbrbZIP27*) regulated the immune signaling of plants downstream of the MPK3 signal transduction pathway [36].

### Conserved motif and structure analyses of pear bZIP proteins

Given that the structure of exon and intron can provide important evidence supporting the phylogenetic relationships of a gene family [37], a rootless phylogenetic tree was established to analyze the evolutionary history of the *PbrbZIP* gene family by multi-sequence alignment (Fig. 2a). In this study, an online program of Multiple Expectation Maximization for Motif Elicitation (MEME) was used to detect motif patterns. As shown in Fig. 2b, 20 preserved patterns, including the bZIP domain (motif #1 and motif #4), were identified, and their multilevel pattern amino acid consensus sequences are listed in Table S2. The proteins categorized within the same group tended to share a similar motif composition, but varied significantly between groups, which further supported the group definitions. As shown in Fig. 2b, among PbrbZIPs, motif #1, containing a basic DNA-binding domain, which belonged to a typical bZIP domain, was detected in all members as a conserved pattern, except PbrbZIP03. Some patterns were present only in specific subgroups, including motif #6 in subgroups I and E; motif #7 in subgroups C, I, S, E, F, G, and M; and motif #17 in subgroups A and UN, except PbrbZIP03. However, some unique patterns could be detected only in specific subgroups. For instance, the pattern [#2, 3, 5, 8, 19] in subgroup D, pattern [#9, 10, 12, 13, 14, 18] in subgroup I, pattern #11 in subgroup F, pattern #16 in subgroup A, and pattern #20 in subgroup G. Many subgroups were composed of certain patterns, but huge differences were found among subgroups. According to the results of gene structure analysis, the number of exons and the gene structure of the *PbrbZIP* gene family were diverse (Fig. 2c). As shown in Fig. 2c, 22 *bZIP* genes were identified with no introns, all of which belonged to subgroups S, F, and *PbrbZIP92*, and which accounted for 23.6% of the total number of *PbrbZIP* genes. Among the intron-containing genes, the number of introns in open reading frames ranged from 0 to 11, and the number of introns in different groups varied greatly. For example, a greater degree of variation in the number of introns occurred in subgroups A, D, and G, ranging from 0 to 4, 7 to 11, and 3 to 11, respectively. However, the number of introns in the remaining groups was smaller, for example, three in subgroups E and H and three to four in subgroup I. As a result, we proposed that exon loss and gain occurred during the evolution of *PbrbZIP* genes, and the evolution and division among different subgroups might occur at an early stage.

### Evolutionary and phylogenetic relationship of *PbrbZIP* genes

**Fig. 3:**
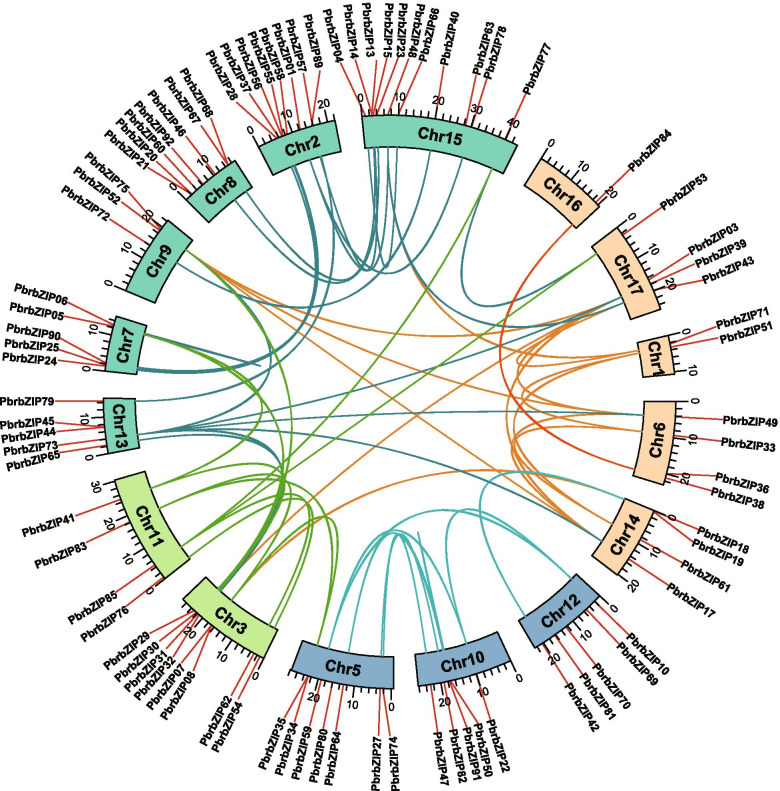
Distribution and collinearity of *PbrbZIPs*. The lines in various colors within the circle indicate collinearity relationships between PbrbZIP genes. The red lines along the circumference of the circle show the location of genes on chromosomes

An intragenomic synteny analysis was performed to understand the evolutionary process of *PbrbZIP* genes, and conservation chromosome blocks were identified in Chinese white pears. In Fig. 3, the landscape of ortholog *PbrbZIP* gene pairs showed that the chromosomal distribution was random. WGD/segmental duplication, tandem duplication, and transposition events are the major causes of gene family expansion and affect the evolution of protein-coding gene families [38]. In this study, duplication events were detected in the *bZIP* gene family, and each gene was assigned to one of five different types of duplications: singleton, dispersed, proximal, tandem, and WGD/segmental through running the MCScanX package. Five types of duplications were all detected causing *PbrbZIP* genes to expand (Tables 2 and S3). The results showed that 65 genes (78.31%) of the *bZIP* gene family of Chinese white pears were duplicated and preserved from segmental/WGD events, and almost 12 *PbrbZIP* genes (14.46%) belonged to the dispersed type.

**Table 2:** Numbers of bZIP genes from different origins in pear (*Pyrus bretschneideri*)

Duplication type	Singleton	Dispersed	Proximal	Tandem	WGD/segmental
**No. of bZIP genes from different origins (percentage)**	1(1.20)	12(14.46)	2(2.41)	3(3.61)	65(78.31)

The Ks value (synonymous substitutions per site) could be used to estimate the dates of WGD and segmental duplication [39].. Previous studies showed that two genome-wide replication events occurred in the pear genome: the ancient WGD occurred in ~ 140 million years ago (MYA) and the modern WGD occurred in 30–45 years of MYA [33, 40]. The Ks values were used to estimate the evolutionary date of gene duplication events in the *PbrbZIP* gene family. As seen in the replication period estimated by Ks values in Table 3, most of the *PbrbZIP* genes were around the recent WGD event, and some were in the ancient WGD. The ratio of nonsynonymous substitutions per nonsynonymous site (Ka) to Ks was also used to predict the selection pressure of duplicated genes: Ka/Ks > 1 meant positive selection, Ka/Ks = 1 meant neutral selection, and Ka/Ks < 1 denoted purification (negative) selection [41]. The Ka/Ks ratio of all *PbrbZIP* genes was lower than 1, indicating that *PbrbZIP* genes evolved mainly under purifying selection.

**Table 3:** The duplicate mode and estimation of absolute date for large-scale duplication events for *PbrbZIPs*

Method	Colinearity gene pairs		Duplication type		Ka	Ks	Ka/Ks	MYA
	Gene1	Gene2	Gene1	Gene2				
NG	*PbrbZIP51*	*PbrbZIP61*	WGD	WGD	0.52	2	0.26	669.26
NG	*PbrbZIP71*	*PbrbZIP48*	WGD	WGD	0.08	0.11	0.72	37.1
NG	*PbrbZIP71*	*PbrbZIP43*	WGD	WGD	Na	Na	Na	Na
NG	*PbrbZIP51*	*PbrbZIP33*	WGD	WGD	0.56	2.83	0.2	947.37
NG	*PbrbZIP51*	*PbrbZIP16*	WGD	NA	0.07	0.23	0.29	78.55
NG	*PbrbZIP50*	*PbrbZIP91*	WGD	WGD	0	0.02	0.07	7.81
NG	*PbrbZIP22*	*PbrbZIP10*	WGD	WGD	0.46	1.74	0.26	581.6
NG	*PbrbZIP22*	*PbrbZIP59*	WGD	WGD	0.06	0.25	0.22	85.02
NG	*PbrbZIP91*	*PbrbZIP64*	WGD	WGD	0.05	0.14	0.35	47.99
NG	*PbrbZIP82*	*PbrbZIP27*	WGD	WGD	0.07	0.19	0.35	63.45
NG	*PbrbZIP47*	*PbrbZIP74*	WGD	WGD	0.09	0.23	0.38	78.38
NG	*PbrbZIP50*	*PbrbZIP64*	WGD	WGD	0.05	0.11	0.42	38.21
NG	*PbrbZIP85*	*PbrbZIP77*	WGD	WGD	0.34	Na	Na	Na
NG	*PbrbZIP85*	*PbrbZIP53*	WGD	WGD	0.35	Na	Na	Na
NG	*PbrbZIP41*	*PbrbZIP30*	WGD	WGD	0.05	0.22	0.24	74.34
NG	*PbrbZIP76*	*PbrbZIP54*	WGD	WGD	0.03	0.16	0.15	54.99
NG	*PbrbZIP85*	*PbrbZIP62*	WGD	WGD	0.03	0.24	0.14	80.94
NG	*PbrbZIP83*	*PbrbZIP08*	WGD	WGD	0.03	0.16	0.22	53.83
NG	*PbrbZIP83*	*PbrbZIP35*	WGD	WGD	0.04	0.15	0.24	50.18
NG	*PbrbZIP41*	*PbrbZIP05*	WGD	WGD	0.25	1.55	0.16	519.43
NG	*PbrbZIP42*	*PbrbZIP19*	WGD	WGD	0.05	0.16	0.3	53.19
NG	*PbrbZIP10*	*PbrbZIP59*	WGD	WGD	0.5	1.61	0.31	539.82
NG	*PbrbZIP73*	*PbrbZIP17*	WGD	WGD	0.64	2.57	0.25	859.53
NG	*PbrbZIP73*	*PbrbZIP39*	WGD	WGD	0.34	1.55	0.22	518.94
NG	*PbrbZIP79*	*PbrbZIP57*	WGD	WGD	0.12	0.19	0.64	62.31
NG	*PbrbZIP65*	*PbrbZIP32*	WGD	WGD	0.06	0.25	0.23	82.12
NG	*PbrbZIP73*	*PbrbZIP31*	WGD	WGD	0.05	0.26	0.19	88.16
NG	*PbrbZIP73*	*PbrbZIP49*	WGD	WGD	0.57	1.76	0.33	588.3
NG	*PbrbZIP73*	*PbrbZIP52*	WGD	WGD	0.36	1.42	0.25	476.29
NG	*PbrbZIP17*	*PbrbZIP39*	WGD	WGD	0.62	Na	Na	Na
NG	*PbrbZIP19*	*PbrbZIP07*	WGD	WGD	0.5	Na	Na	Na
NG	*PbrbZIP17*	*PbrbZIP49*	WGD	WGD	0.15	0.3	0.49	98.8
NG	*PbrbZIP61*	*PbrbZIP33*	WGD	WGD	0.11	0.21	0.52	71.39
NG	*PbrbZIP17*	*PbrbZIP52*	WGD	WGD	0.6	2.47	0.25	824.95
NG	*PbrbZIP04*	*PbrbZIP66*	WGD	WGD	0.01	0.01	0.9	3
NG	*PbrbZIP48*	*PbrbZIP43*	WGD	WGD	0.08	0.11	0.72	37.1
NG	*PbrbZIP77*	*PbrbZIP53*	WGD	WGD	0.1	0.31	0.34	102.63
NG	*PbrbZIP78*	*PbrbZIP01*	WGD	WGD	0.08	0.2	0.38	67.79
NG	*PbrbZIP48*	*PbrbZIP01*	WGD	WGD	0.61	1.14	0.54	381
NG	*PbrbZIP15*	*PbrbZIP89*	WGD	WGD	0.27	1.57	0.17	524.43
NG	*PbrbZIP13*	*PbrbZIP67*	WGD	WGD	0.03	0.2	0.13	66.97
NG	*PbrbZIP23*	*PbrbZIP46*	WGD	WGD	0.09	0.23	0.38	77.05
NG	*PbrbZIP40*	*PbrbZIP72*	WGD	WGD	0.03	0.19	0.17	62.26
NG	*PbrbZIP13*	*PbrbZIP11*	WGD	NA	0.07	0.31	0.23	102.58
NG	*PbrbZIP84*	*PbrbZIP38*	WGD	WGD	0.04	0.16	0.21	55.06
NG	*PbrbZIP39*	*PbrbZIP31*	WGD	WGD	0.31	1.44	0.22	481.17
NG	*PbrbZIP39*	*PbrbZIP49*	WGD	WGD	0.52	Na	Na	Na
NG	*PbrbZIP39*	*PbrbZIP52*	WGD	WGD	0.06	0.2	0.29	66.62
NG	*PbrbZIP37*	*PbrbZIP90*	WGD	WGD	0.01	0.14	0.09	45.83
NG	*PbrbZIP56*	*PbrbZIP25*	WGD	WGD	0.1	0.24	0.42	81.4
NG	*PbrbZIP55*	*PbrbZIP24*	WGD	WGD	0.05	0.21	0.26	69.07
NG	*PbrbZIP58*	*PbrbZIP26*	dispersed	NA	0.04	0.14	0.27	47.62
NG	*PbrbZIP08*	*PbrbZIP35*	WGD	WGD	0	0.01	0.2	2.81
NG	*PbrbZIP07*	*PbrbZIP34*	WGD	WGD	0.01	0.01	1.09	3.77
NG	*PbrbZIP30*	*PbrbZIP05*	WGD	WGD	0.26	1.74	0.15	581.11
NG	*PbrbZIP31*	*PbrbZIP52*	WGD	WGD	0.34	1.35	0.25	450.63
NG	*PbrbZIP49*	*PbrbZIP52*	WGD	WGD	0.58	Na	Na	Na
NG	*PbrbZIP05*	*PbrbZIP06*	WGD	WGD	Na	Na	Na	Na
NG	*PbrbZIP06*	*PbrbZIP09*	WGD	NA	0.02	0.15	0.11	48.73
NG	*PbrbZIP05*	*PbrbZIP09*	WGD	WGD	0.02	0.15	0.11	48.73
NG	*PbrbZIP67*	*PbrbZIP11*	WGD	WGD	0.06	0.34	0.19	114.28
NG	*PbrbZIP60*	*PbrbZIP86*	dispersed	NA	0.03	0.08	0.36	26.09
NG	*PbrbZIP86*	*PbrbZIP87*	WGD	NA	Na	Na	Na	Na

### Expression patterns of *PbrbZIP* genes in response to cold stress

**Fig. 4:**
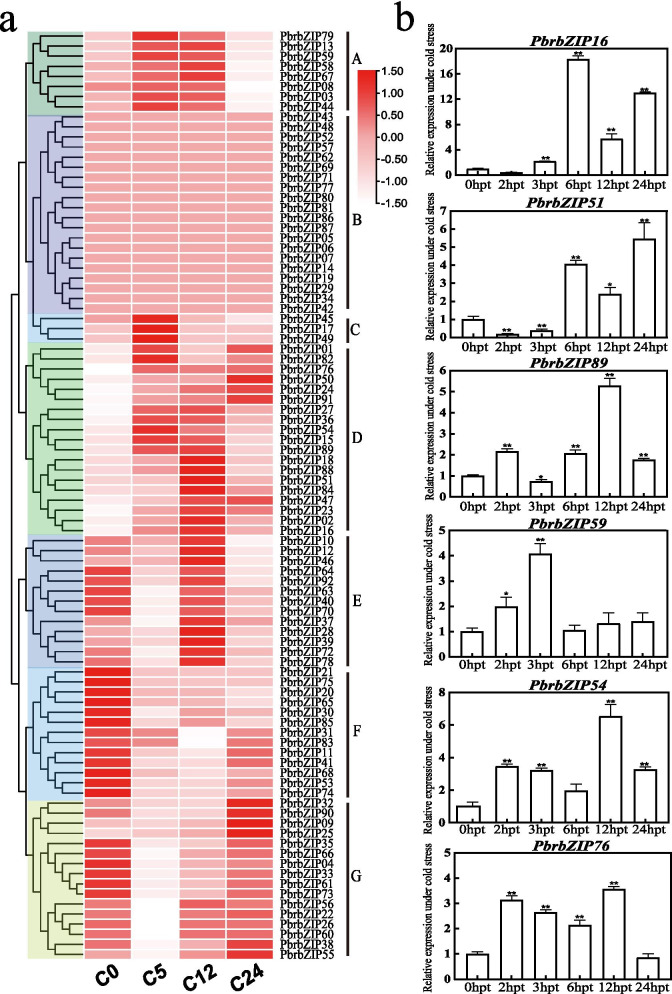
Expression profile and expression analysis of *PbrbZIPs* under cold stress. **a** Expression profile of *PbrbZIPs* under cold stress; **b** Relative expression of PbrbZIP16, PbrbZIP51, PbrbZIP89, PbrbZIP59, PbrbZIP54 and PbrbZIP76 with cold treatment. The pear tubulin was used as internal reference for the normalization. The statistical analyses were performed using student’s t-test (* *p* < 0.05, ** *p* < 0.01)

**Fig. 5:**
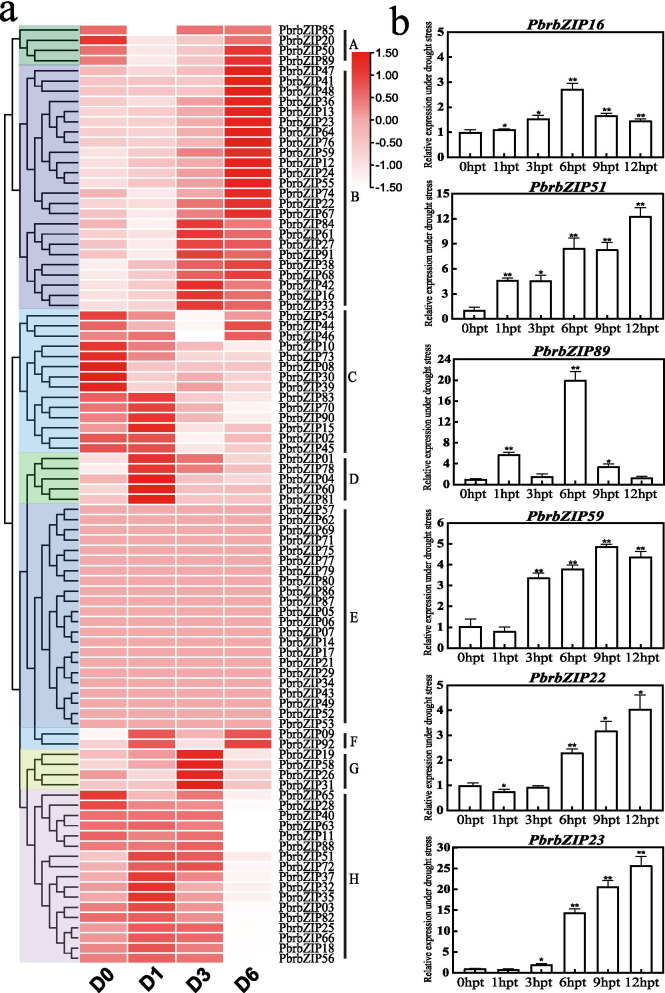
Expression profile and analysis of *PbrbZIPs* under drought stress. **a** Expression profile of *PbrbZIPs* under drought stress; **b** Relative expression *PbrbZIP16*, *PbrbZIP51*, *PbrbZIP89*, *PbrbZIP59*, *PbrbZIP22* and *PbrbZIP23* with drought treatment. The pear tubulin was used as internal reference for the normalization. The statistical analyses were performed using student’s t-test (* *p* < 0.05, ** *p* < 0.01)

The bZIP proteins might be related to cold and drought stresses in plants [22, 42, 43]. However, limited information regarding the response of bZIP TFs to drought and cold stresses has been reported in Chinese white pears. The response of pears to drought and low-temperature stresses was studied by analyzing the transcriptome data of *PbrbZIP* genes (Figs. 4a and 5a).

In Fig. 4a, cluster A contained eight *PbrbZIP* genes that were significantly upregulated after cold treatment at 5 h post treatment (hpt) and 12 hpt. Cluster C with three genes experienced upregulation at 5 hpt and then began a downregulation between 12 hpt and 24 hpt. Again, 19 genes of cluster D were highly induced from 5 hpt to 24 hpt. Most genes of clusters E, F, and G were highly expressed at 0 hpt and downregulated at 5 hpt, but then upregulated again later. Most genes in cluster B were not significantly induced by cold treatment at all tested times. Twenty-three genes that were at least 1.5 times more regulated after cold treatment were chosen to investigate their expression patterns (Table S4). Six genes were found in subgroup I, five genes in subgroup S, three genes in A and G, and two genes in C and F; *PbrbZIP36* belonging to subgroup K were upregulated under cold stress. The expression levels of 19 genes in cluster D were higher than those in other clusters, and most genes in cluster B belonged to S, G, I, and F subgroups. These results indicated that genes of A, C, G, I, and S subgroups mainly mediated cold stress responses by taking part in biological pathways.

Quantitative real-time polymerase chain reaction (qRT-PCR) were performed to analyze the relative transcript abundance of six selected genes so as to confirm whether the expression of these genes differed under low-temperature stress. As shown in Fig. 4b, *PbrbZIP54*, *PbrbZIP76*, and *PbrbZIP89* from subgroup S were upregulated at 2 hpt but downregulated at 3 hpt, and upregulated again at 12 hpt. *PbrbZIP16* and *PbrbZIP51* in subgroup G were upregulated at 6 and 24 hpt but downregulated at 12 hpt. *PbrbZIP59* belonging to subgroup C was upregulated only at 3 hpt after cold treatment and then downregulated. These results closely matched the RNA-seq data.

### Expression patterns of *PbrbZIP* genes in response to drought stress

The same approach was used to analyze bZIP TFs in response to drought stress. As shown in Fig. 5a, cluster A (four genes) was significantly downregulated after drought treatment for 1 and 3 hpt and upregulated at 6 hpt. Cluster B contained 24 *PbrbZIP* genes that were upregulated at 6 hpt. In cluster C, 14 *PbrbZIP* genes were downregulated at 3 hpt and 6 hpt. Cluster D contained five genes upregulated at 1 hpt after drought treatment. Cluster E (22 genes) had no apparent differences in expression in response to drought stress. Cluster F contained two genes that were significantly upregulated at 1 and 6 hpt, but downregulated at 3 hpt. Four genes in cluster G were upregulated at 3 hpt, but downregulated at 6 hpt under drought stress. The genes belonging to cluster H had relatively high expression from 0 to 3 hpt, but downregulated at 6 hpt. Nineteen genes, which were upregulated at least twofold under drought stress, were selected for a further survey of their expression patterns (Table S4). Five genes in group I, four genes in group S, three genes in group C, two genes in A and G, and one gene in B, F, and K were upregulated under drought stress. Compared with the genes in other clusters, seven genes in cluster B and two genes in cluster A were more significantly upregulated at 3 hpt. Three genes belonged to subgroup C, two genes to subgroup G, and one gene to subgroups B, K, and S. Therefore, the *PbrbZIP* genes from these subgroups might be involved in some biological processes to improve the drought tolerance of pears. Meanwhile, seven genes, including *PbrbZIP89*, *PbrbZIP76*, *PbrbZIP36*, *PbrbZIP16*, *PbrbZIP51*, *PbrbZIP59*, and *PbrbZIP24*, were found to be strongly upregulated after drought treatment as well as under cold treatment. The qRT-PCR results closely matched the RNA-seq data of this study (Fig. 5b). The expression of all chosen genes peaked at 6, 9, and 12 hpt, and then began to decline. These results indicated that all the aforementioned genes had a significant response to drought and cold temperature treatment. Moreover, the expression patterns of *PbrbZIP16* and *PbrbZIP51* indicated that these genes were involved in stress resistance and specific genes might have different response patterns under different stresses.

### Silencing *PbrbZIP51* in *P. betulaefolia* provided sensitivity to drought stress

**Fig. 6:**
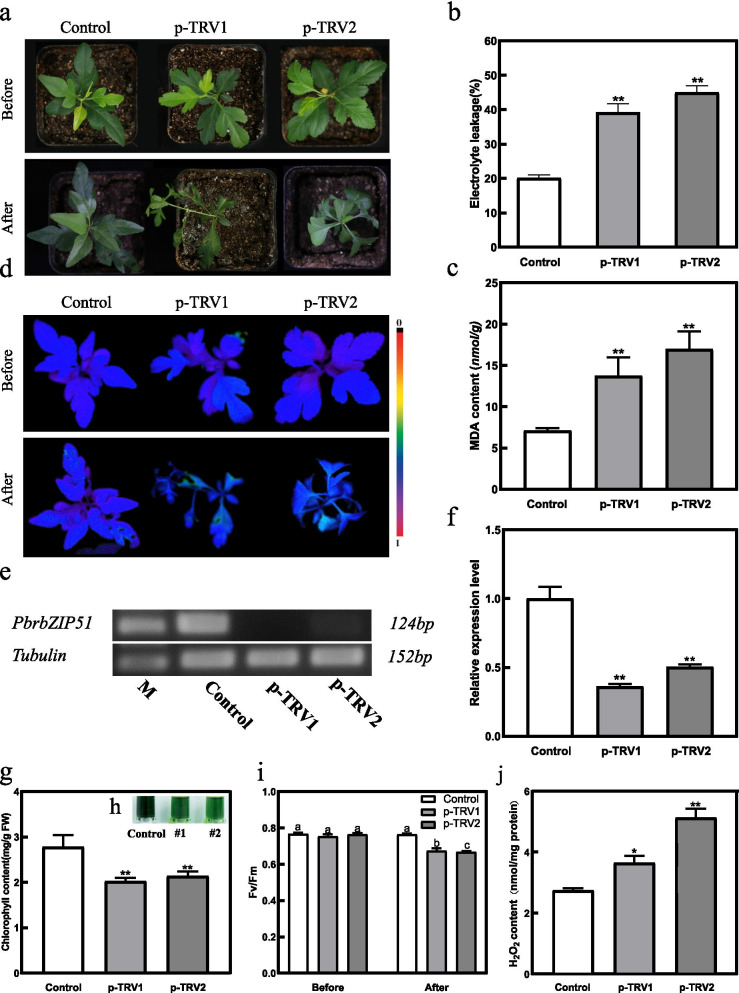
Analysis of drought tolerance in the *PbrbZIP51*-silenced *Pyrus betulaefolia* plants. Phenotype of 1-month-old *PbrbZIP51*-silenced plants before and after drought treatment for 15 days (**a**). Electrolyte leakage (EL) (**b**). Malondialdehyde (MDA) concentrations after drought treatment (**c**). Chlorophyll fluorescence imaging of silenced plants and control plants (**d**). The expression of *PbrbZIP51* was detected by RT-PCR (**e**) and qRT-PCR (**f**) at 3 days after the injection. Chl content of control and pTRV-*PbrbZIP51* silencing plants (pTRV-1, pTRV-2) at the end of the drought stress (**g**), and the phenotype (**h**) of control and pTRV-*PbrbZIP51* silencing plants after drought treatment, and the Fv/Fm ratios (**i**). Quantitative measurement of H_2_O_2_ levels after drought treatment (**j**)

As a significantly upregulated gene under both cold and drought stresses, *PbrbZIP51* was selected to perform virus-induced gene silencing (VIGS) to further explore the role of *PbrbZIP* genes in drought tolerance. As shown in Fig. 6a–d, VIGS plants (p-TRV1 and p-TRV2) suffered more severe damage than control after drought treatment for 15 days. As shown in Fig. 6e and f, the expression of *PbrbZIP51* was suppressed in silenced seedlings. The electrolyte leakage (EL) (Fig. 6b) and malondialdehyde (MDA) Fig. 6c) concentrations were significantly higher in silenced pear seedlings than in control seedlings. Chl fluorescence in silenced plants was suppressed, with significantly lower Fv/Fm ratio and Chl content compared with that in control plants (Fig. 6g–i). Furthermore, quantitative measurements of the H_2_O_2_ content showed that the H_2_O_2_ content of the silenced plants was much higher than those of the control plants (Fig. 6j). These results suggested that the *PbrbZIP51* gene was silenced to enhance the sensitivity to drought in *P. betulaefollia*.

## Discussion

Cold and drought stresses are two of the most important limiting environmental factors that can seriously impair crop productivity. TFs play an important role in protecting plants from stress-related damage by regulating the expression levels of downstream target genes [44]. Therefore, genetic engineering of TFs involved in stress resistance has been proposed to be a robust strategy for improving the stress tolerance of crop plants [45, 46]. Since the release of genome sequencing data from Chinese white pears, many *TF* genes have been identified and characterized at the genome-wide level, such as *NAC TFs* (183 genes), *PbBAMs* (17), and *PbrbHLH* genes (197) in pears [47–49].

As a large family in plant TFs, *bZIP* genes have been found to be involved in several important biological activities [7]. However, the *PbrbZIP* family has not been studied in much detail in pears, and the *PbrbZIP* family genes in pears have been rarely studied. In this study, 92 *PbrbZIP* genes were identified in Chinese white pears and classified into 14 subgroups based on the phylogenetic analysis, gene structure, and protein conserved motif analysis. Subgroup S had the largest number of *PbrbZIP*s, followed by subgroups I and A. Subgroup K had the least *PbrbZIP*s. These results were similar to the case in *A. thaliana* [26]. Both intron/exon organizations and protein motif patterns were too diverse according to the analysis of gene and protein structure in the *PbrbZIP* family. Despite the conserved distribution pattern for exons and untranslated regions (UTRs) in subgroups C, D, E, G, I, and S, many other subgroups exhibited diversity in exon number and structure, consistent with the results of protein pattern analysis. The 20 preserved motifs detected by the online MEME program indicated that the subgroup division of the *PbrbZIP* gene family might have occurred at an earlier stage, and *PbrbZIP* genes might have played multiple roles in the evolutionary process of adaptation to environmental stresses.

The results of gene duplication analysis showed that WGD/segment events drove the expansion of the *PbrbZIP* gene family. Sixty-five *PbrbZIP* genes (78.31%) were categorized into the WGD/segmental type, and 12 genes (14.46%) belonged to the dispersed type, which might be due to the high ratio of self-incompatibility and the domestication process of pears. WGD/segment and dispersed duplications played an essential role in expanding the pear *bZIP* gene family from the aforementioned results. Based on the estimated results of Ks, *PbrbZIP* genes were duplicated around the time of the most recent WGD event and some from ancient WGD. The Ka/Ks ratios showed that the *PbrbZIP* genes evolved primarily through purifying selection.

Function enrichment analyses showed that *PbrbZIP* genes were primarily enriched in functions and processes closely related to TFs, and the pathways they categorized were the principal mechanisms by which bZIP family TFs regulated gene expression downstream, such as hormone signal transduction pathways, circadian rhythm, and MAPK signaling.

Based on the previous transcriptome profile, most *PbrbZIP* genes were found to be significantly induced by stress treatments. Twenty-three *PbrbZIP* genes upregulated under cold treatment and 19 differently expressed *PbrbZIP* genes under drought treatment were detected. In addition, some genes in groups A, C, G, I, and S were possibly involved in biological pathways of drought and cold stress responses. *PbrbZIP* genes were analyzed using qRT-PCR analysis under stress treatments to verify whether *PbrbZIP* genes were involved in response to cold or drought stresses. The results showed that the expression of all tested genes was significantly altered under drought or cold treatments. The expression pattern of the same gene between two treatments could be diverse. For instance, under cold treatment, the expression of *PbrbZIP59* showed an upregulated trend at first before being downregulated. However, under drought stress, it was downregulated first and then upregulated. *PbrbZIP16* was more intensively upregulated under cold conditions than under drought stress. In addition, *PbrbZIP51*, a highly upregulated gene induced under drought stress conditions, has significantly reduced drought tolerance for RNAi pear seedlings. These results indicated that *PbrbZIP* genes played a role in response to drought and cold stresses in pears, and the processes they were involved in seemed different under various stress conditions. The bZIP TF played an important role in plant regulation and development through protein–protein interactions with variable elements; moreover, the specific functions of genes were realized through the dimer formed by the specific interaction between the monomeric bZIP forms [50, 51]. However, how the *PbrbZIP* genes play an important role in the resistance to stress-related injury by regulating the expression level of downstream target genes is still unclear, and the underlying molecular mechanisms require further investigation.

In this study, first the *PbrbZIP* genes were identified, and subsequently their evolutionary relationship and expression patterns were analyzed under abiotic stresses in pears. *PbrbZIP*s were involved in the drought and cold stress tolerance pathways by the analyses of qRT-PCR, and the functional analysis indicated that *PbrbZIP51* played essential roles in drought stress tolerance in pears. Other genes need to be tested for tolerance to cold and drought stresses in future studies. The results of this study provided a basis for genetic engineering screening of new candidate *bZIP* genes in pear cultivars with stress tolerance.

## Conclusions

A total of 92 *PbrbZIP* genes were identified from the pear genome, which were divided into 14 subgroups based on the results of protein motifs and intron/exon characteristics and phylogenetic analysis. The recent WGD (~ 30–45 MYA) and dispersed duplications may be the main driving force for the large-scale amplification of the *bZIP* gene family in Chinese white pears. The large-scale amplification of genes in the *PbrbZIP* family has been proven to be driven by purifying selection. Besides, transcriptome sequencing profile, analyses of qRT-PCR, and VIGS indicated that *PbrbZIP* genes might play a vital role in response to drought and cold stresses, and the pathway they participated in might differ in response to drought and cold stresses. These results may be useful in developing strategies to increase tolerance further to stress in pears, and providing a foundation for advanced studies to evaluate the mechanisms of *bZIP* gene tolerance to cold and drought stresses in pears.

## Methods

### Plant materials and bacterial strains

Pear seeds (*Pyrus.betulifolia*) were obtained from our pear germplasm orchard of the Center of Pear Engineering Technology Research situated at Hushu in Nanjing. Pear seedlings were grown in a greenhouse with 16 h/8 h light/dark photoperiod, 75% relative humidity and 25 °C. *Agrobacterium tumefaciens* GV3101 was grown in LB media supplemented with kanamycin and Rif at 28 °C in an orbital shaker at 200 rpm and harvested during the log phase of growth for infiltration.

### Identification of bZIP genes

The whole-genome sequence of Chinese White pears along with GFF3 (general feature format file) was derived from (http://peargenome.njau.edu.cn), and the seed files of bZIP conserved domain (PF00170, PF07716 and PF07777) were downloaded from the Pfam database (http://pfam.sanger.ac.uk/). The conserved Pfam domain was detected by running the Hidden Markov Model (HMM) software, E-value< 0.05 [52]. Additionally, online SMART program (http://smart.embl-heidelberg.de/) and NCBI Batch CD-search tool were used to detect the existence of the conserved bZIP domain in each protein sequence [53]. The annotation information of the Chinese white pear genome was fetched from the GFF file, and the R script was used to display the result. The published bZIP protein sequence of *A. thaliana* was downloaded from the TAIR database (http:// www.arabidopsis.org/).

### Sequence and phylogenetic analyses

We imported the pear and *A. thaliana* bZIP protein sequences into MEGA 7 software (http://www.megasoftware.net/) [54] and used ClustalW for multiple sequence alignments. The Neighbor-Joining (NJ) phylogenetic tree was constructed by using MEGA 7 software with the bootstrap set to 1000. P-distance and pairwise deletion which is one of the optional parameters were considered. The annotation and review of the phylogenic trees were completed by iTOL (https://itol.embl.de/) and EvolView (https://www.evolgenius.info/evolview/).

### Gene structure and conserved motif analyses

Conserved motif analysis was performed by online Multiple Expectation Maximization for Motif Elicitation (MEME) [55] (http://meme.ebi.edu.au/meme/ intro.html) with default parameters, and maximum number of motifs parameter set as 20. The intron/exton structures analysis of 92 *PbrbZIP* genes was found through general feature format (GFF3) files and visualized by using Gene Structure Display Server [56] (http://gsds.cbi.pku.edu.cn/).

### Chromosomal localization and synteny analyses

The chromosome location information was taken from the GFF file. The synteny among *PbrbZIPs* was analyzed using the same procedure used in the PGDD (http://chibba.agtec.uga.edu/duplication/). Primarily, local all-vs-all BLASTP research among the identified *PbrbZIP* genes was carried out (E < 1e ^− 10^). Later, MCScanX was used for the determination of syntenic gene pairs with the BLASTP result and gene location information used as input files [57]. Singleton, dispersed, proximal, tandem and WGD/segmental duplications of *PbrbZIPs* were identified by employing the downstream analysis tool (duplicate_gene_classifier) in the MCScanX package. Results were displayed with the circos-0.69 software [58]. The Ka and Ks values were analyzed using KaKs-calculator 2.0 [59]. The date of segmental duplication events was estimated by using the mean Ks value which considered the succeeding pairs of homologous genes within 100 Kb on each side of the *PbrbZIPs*.

### Expression analysis of *PbrbZIPs* under cold and drought stress conditions

Published transcriptomic data (FPKM values) characterizing the total RNA of drought treatment samples, including D0, D1, D3, D6 (harvested at 0 hpt, 1 hpt, 3 hpt and 6 hpt under drought stress) were downloaded from Li et al. (2016) [60]. Cold treatment samples, including C0, C5, C12, C16 (harvested at 0 hpt, 5 hpt, 12 hpt and 24 hpt under cold stress) were downloaded from Yang and Huang (2018) [61]. The expression patterns of *PbrbZIPs* under drought and cold stress were determined, and the differentially expressed genes were identified with the threshold |log2^FC^| > 1. These results were visualized by TBtools v1.082 [62].

For the expression analysis, nine-week-old pear seedlings, exposed to drought and cold stress, were used to test the relative transcript level of selected genes by qRT-PCR. The seedlings were placed in a chamber set at 4 °C for 0 hpt, 2 hpt, 3 hpt, 6 hpt, 12 hpt and 24 hpt. For drought treatment, the seedlings were placed on dry filter papers for 0 hpt, 1hpt, 3 hpt, 9 hpt, 12 hpt and 24 hpt under ambient environment. The total RNA of the pear was extracted as instructed in RNA kit (Tiangen, Beijing, China), and the cDNA was synthesized using PrimeScript RT (Trans Gen) reagent kit. NCBI online tool Primer-BLAST (https://www.ncbi.nlm.nih.gov/tools/ primer-blast/index.cgi? LINK LOC=Blast Home) was used to design the specialized primers of the constitutive *TUB* (*Pbr042345.1*, as internal control) [63] and eight tested *PbrbZIPs* (Table S5). As previously reported, we used SYBR® Green Premix kit (TaKaRa Biotechnology, Dalian, China) to perform qRT-PCR on a Lightcycler480 (Roche), and the PCR mixture was composed of 10 μl 2 SYBR PremixExTaq™, 2.5 μl per primer and 1 μl cDNA model in a final volume of 20 μl [48]. 2^−ΔΔCt^ method was used to evaluate the expression of *PbrbZIPs* under cold and drought stress conditions.

### Generation of silent plants and physiological analyses

As previously reported, the expression of *PbrbZIP51* was suppressed by virus-induced gene silencing (VIGS) -mediated method [47, 64]. Non-injected leaves of each plant were collected and submitted to genomic PCR and qRT-PCR to analyze whether *PbrbZIP51* was silenced after 3 days, and the silenced plants exhibiting similar magnitude of *PbrbZIP51* suppression were used for further drought treatment. Electrolyte Leakage was measured by conductivity monitor according to prior method [65]. Chlorophyll was extracted and analyzed in accordance with prior method [66]. MDA, H_2_O_2_ and O_2_^−^ content were measured by specific analytical kits (Nanjing Jiancheng Bioengineering Institute, Nanjing, China). The level of the chlorophyll fluorescence was measured by Imaging PAM CHL fluorometer. The detail parameters and the estimate method of Fv/Fm values were described by Woo et al. (Walz, Germany) [67].

### Statistical analysis

In this study, abiotic stresses and qRT-PCR expression pattern data were repeated a minimum of three times. The data in the figures were presented in the form of an average ± standard error (SE). All data was analyzed by T-test function in R-language to test the significance level of data between the treatment and the control groups (*P < 0.05, **P < 0.01).

## Supplementary Information

**Additional file 1 **: **Figure S1.** Phylogenetic tree of 78 AtbZIPs and the 92 PbrbZIPs proteins. The phylogenetic tree based on the protein sequences was built by MEGA 7. The annotation and review of the phylogenic tree was completed by EvolView (https://www.evolgenius.info/evolview/).

**Additional file 2 **: **Figure S2.** Functional annotation enrichment analysis. Term enrichment analysis of PbrbZIP proteins. (b) KEGG enrichment analysis of PbrbZIP proteins.2.

**Additional file 3 **: **Figure S3.** Molecular identification of *Pbrbzip51*-silenced pear and other original images of Fig.6**.** Semi-quantitative RT-PCR analysis identification of the plants used specific primers of *PbrbZIP51*. M, DNA marker (DL 2000); Control, untransformed plants. Numbers on the top of the gel panels indicate the *PbrbZIP51*-silenced lines.

**Additional file 4 **: **Table S1.** Detailed characteristics of *PbrbZIPs*.

**Additional file 5 **: **Table S2.** Sequence information of 20 detected motifs in MEME analysis.

**Additional file 6 **: **Table S3.** Duplication type of PbrbZIP genes in pear (*Pyrus bretschneideri*).

**Additional file 7 **: **Table S4.** Differentially expressed genes after stress treatments.

**Additional file 8 **: **Table S5.** Primers of *PbrbZIPs* for qRT-PCR and vector construction.

## Data Availability

All needed genome sequences and genome annotation files of Chinese white pear were obtained from the Nanjing Agricultural University pear genome project website (http://peargenome.njau.edu.cn), and the published bZIP sequences of *A. thaliana* were acquired from the TAIR database (http://www.arabidopsis.org/). The cold transcriptome sequencing data used in this study was got from the additional files from previous report (10.1016/j.gene.2018.03.067). The drought transcriptome sequencing raw data used in this study has been uploaded to the NCBI (https://www.ncbi.nlm.nih.gov/Traces/study/?acc= SRP148620). All databases in this study are available to the public.

## Abbreviations

bZIP: Basic region-leucine zipper
TF: Transcription factor
Abscisic Acid: ABA
AsA: Ascorbic acid
ROS: Reactive oxygen species
ABRE: ABA responsive element
PI: Protein isoelectric points
GRAVY: Grand average of hydropathy
SMART: Simple Modular Architecture Research Tool
NCBI: National Center for Biotechnology Information
Ks: Synonymous substitutions per site
Ka: Nonsynonymous substitutions per nonsynonymous site
hpt: Hours post treatment
EL: Electrolyte leakage
MAPK: Mitogen-activated protein kinase
MDA: Malondialdehyde
HMM: Hidden Markov Model
GSDS: Gene Structure Display Server
MEME: Multiple Expectation Maximization for Motif Elicitation
NJ: Neighbor-Joining
UTR: Untranslated regions
WGD: Whole-genome duplications
MYA: Millions of years Ago
qRT-PCR: Quantitative real-time polymerase chain reaction
VIGS: Virus-induced gene silencing
ORF: Open reading frame
MES: 2-(Nmorpholino) ethanesulfonic acid
