# Supplementary material for: Genome-wide identification and expression analysis of the bZIP transcription factors, and functional analysis in response to drought and cold stresses in pear (Pyrus breschneideri)
Source: BMC Plant Biol. 2021 Dec 9;21:583. doi: 10.1186/s12870-021-03356-0 (PMC8656046; doi:10.1186/s12870-021-03356-0)

Genome-wide identification and expression analysis of the bZIP transcription factors, and functional analysis in response to drought and cold stresses in pear (*Pyrus breschneideri*)

Ming Ma[1,2], Qiming Chen[1,2], Huizhen Dong, Shaoling Zhang* and Xiaosan Huang*

Fig S1 Phylogenetic tree of 78 *AtbZIPs* and the 92 *PbrbZIPs*.

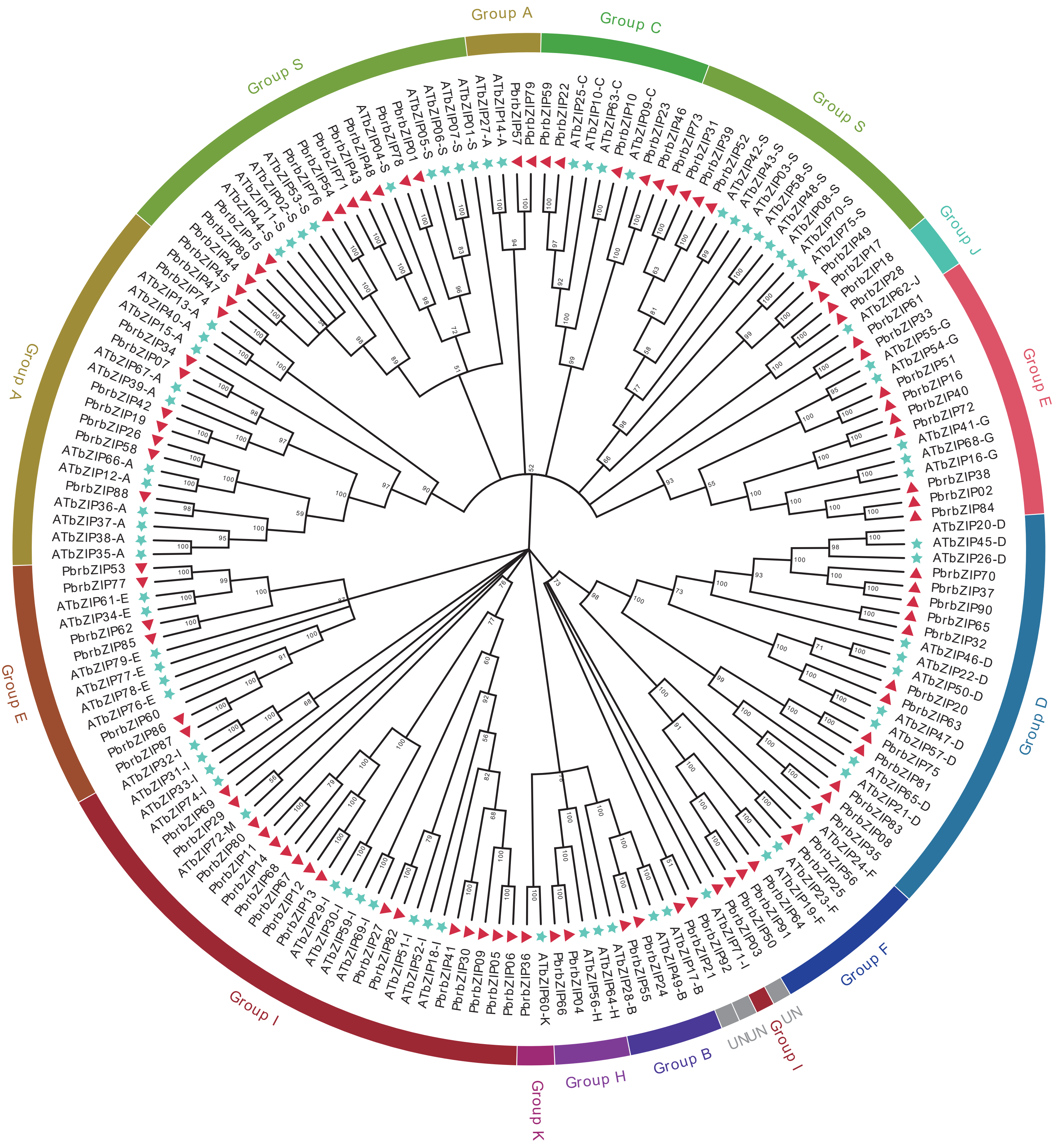

Supplement: Supplementary file 1 — Additional file 1 : Figure S1. Phylogenetic tree of 78 AtbZIPs and the 92 PbrbZIPs proteins. The phylogenetic tree based on the protein sequences was built by MEGA 7. The annotation and review of the phylogenic tree was completed by EvolView (https://www.evolgenius.info/evolview/). [file 12870_2021_3356_MOESM1_ESM.pdf]
