# Supplementary material for: Genome-wide identification and expression analysis of the bZIP transcription factors, and functional analysis in response to drought and cold stresses in pear (Pyrus breschneideri)
Source: BMC Plant Biol. 2021 Dec 9;21:583. doi: 10.1186/s12870-021-03356-0 (PMC8656046; doi:10.1186/s12870-021-03356-0)

Genome-wide identification and expression analysis of the bZIP transcription factors, and functional analysis in response to drought and cold stresses in pear (*Pyrus breschneideri)*

Ming Ma[1,2], Qiming Chen[1,2], Huizhen Dong, Shaoling Zhang* and Xiaosan Huang*

Fig S2 Functional annotation enrichment analysis.

a

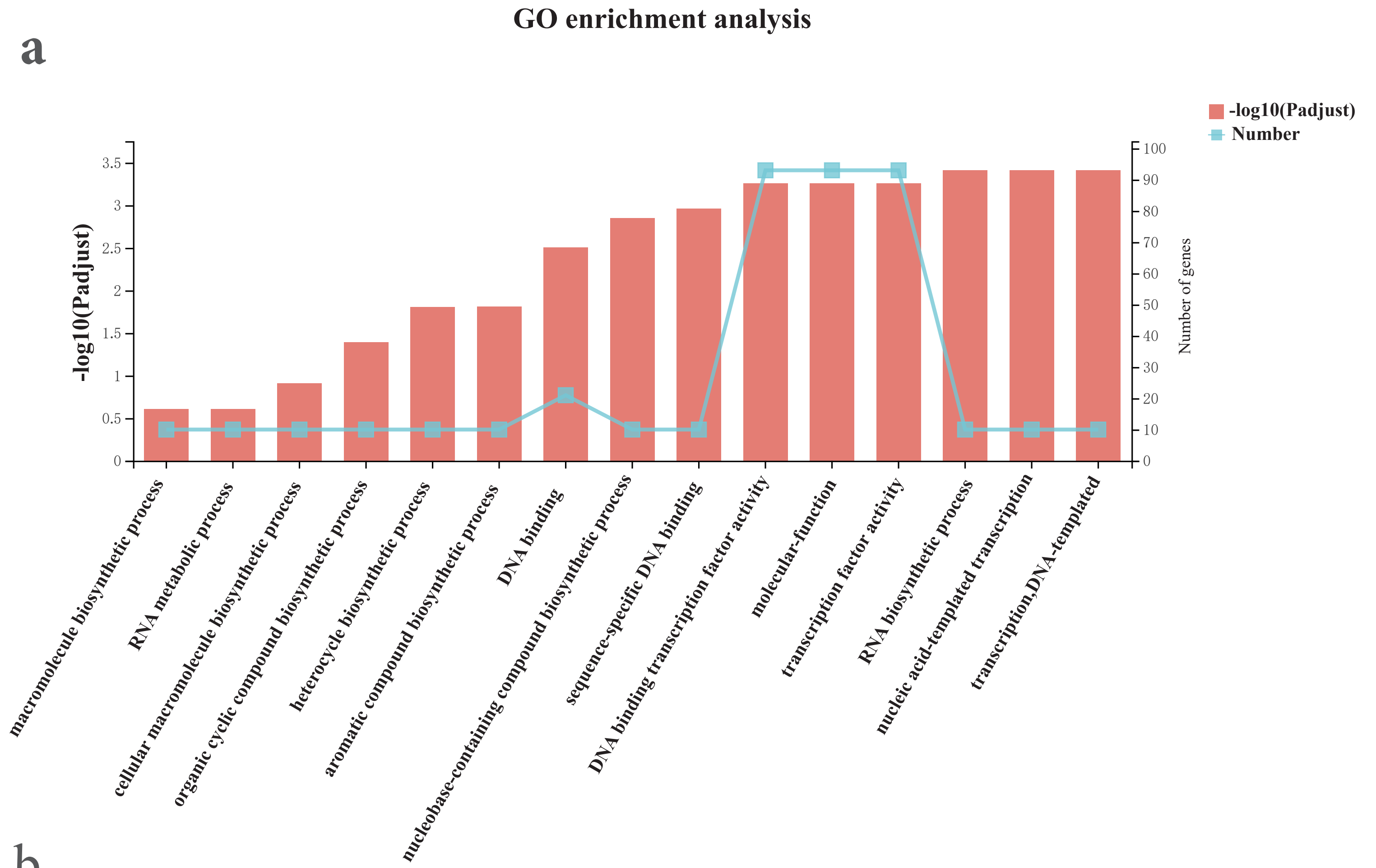

b

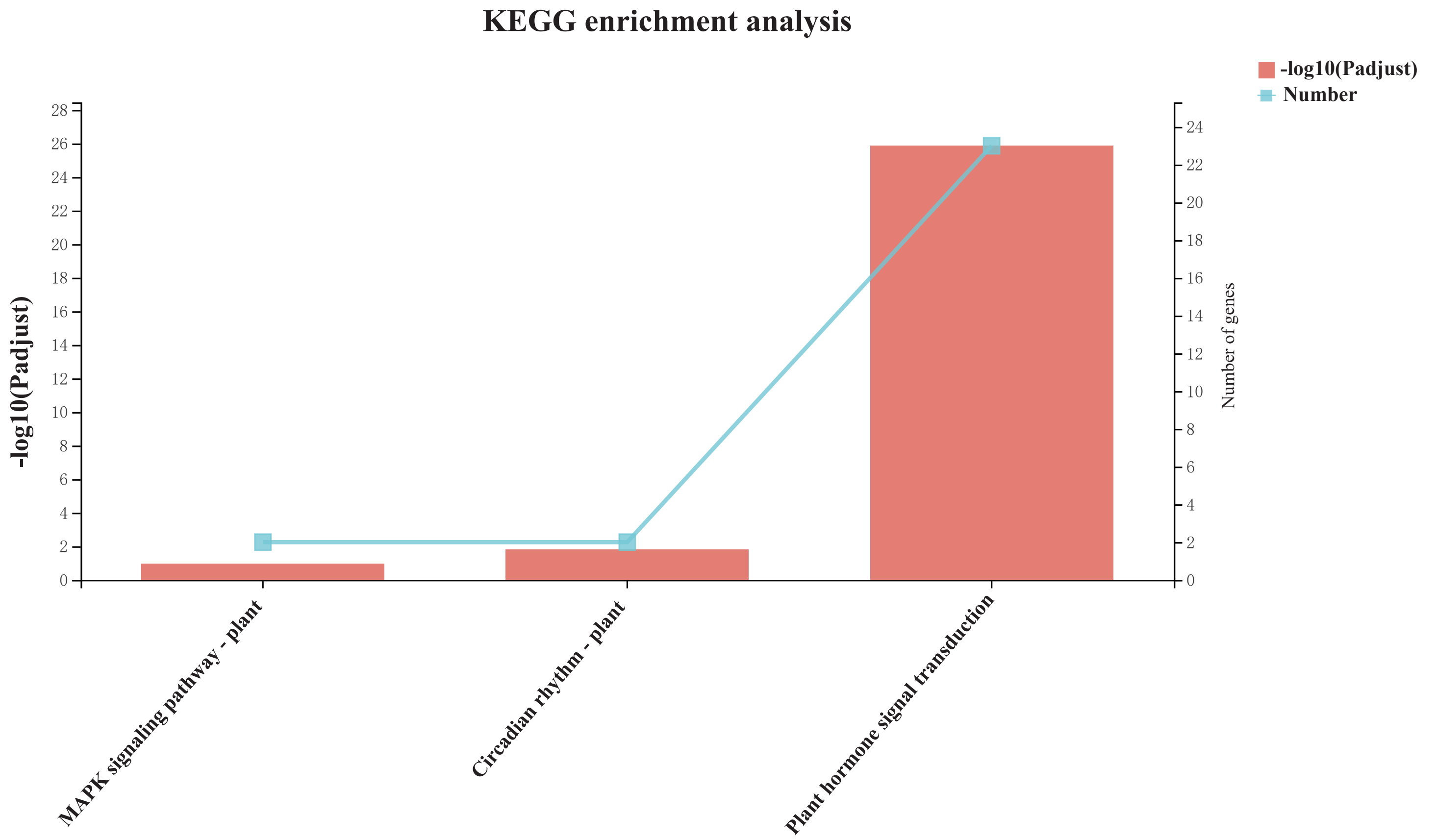

Supplement: Supplementary file 2 — Additional file 2 : Figure S2. Functional annotation enrichment analysis. Term enrichment analysis of PbrbZIP proteins. (b) KEGG enrichment analysis of PbrbZIP proteins.2. [file 12870_2021_3356_MOESM2_ESM.pdf]
