# Supplementary material for: Genome-wide identification and expression analysis of the bZIP transcription factors, and functional analysis in response to drought and cold stresses in pear (Pyrus breschneideri)
Source: BMC Plant Biol. 2021 Dec 9;21:583. doi: 10.1186/s12870-021-03356-0 (PMC8656046; doi:10.1186/s12870-021-03356-0)
Supplement: Supplementary file 3 — Additional file 3 : Figure S3. Molecular identification of Pbrbzip51-silenced pear and other original images of Fig.6. Semi-quantitative RT-PCR analysis identification of the plants used specific primers of PbrbZIP51. M, DNA marker (DL 2000); Control, untransformed plants. Numbers on the top of the gel panels indicate the PbrbZIP51-silenced lines. [file 12870_2021_3356_MOESM3_ESM.pdf]

Genome-wide identification and expression analysis of the bZIP transcription factors, and functional analysis in response to drought and cold stresses in pear (*Pyrus breschneideri)*

Ming Ma[1,2], Qiming Chen[1,2], Huizhen Dong, Shaoling Zhang* and Xiaosan Huang*

Fig S3 Molecular identification of *Pbrbzip51*-silenced pear and other original images of Fig.6.

Fig S3.1 Molecular identification of *Pbrbzip51*-silenced pear.

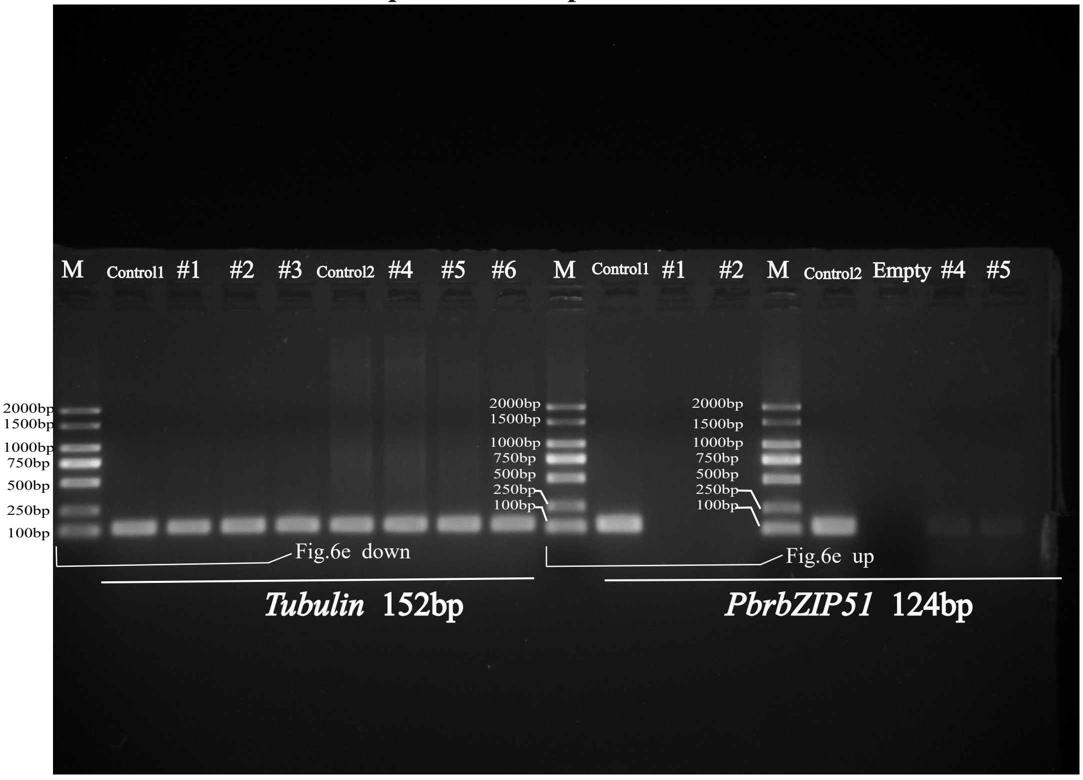

Fig S3.2 Other original images of Fig.6.

Control p-TRV1 p-TRV2

Before

After

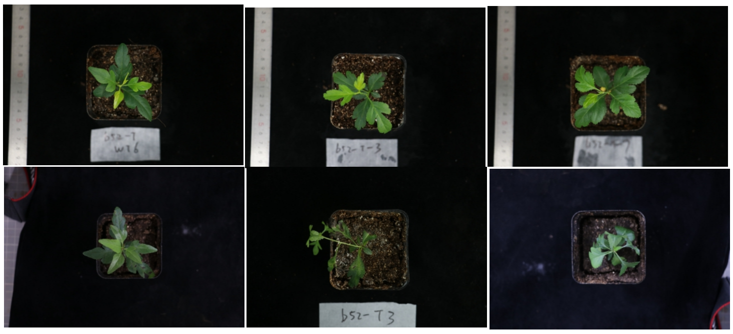

Fig.6a

Control p-TRV1 p-TRV2

Before

After

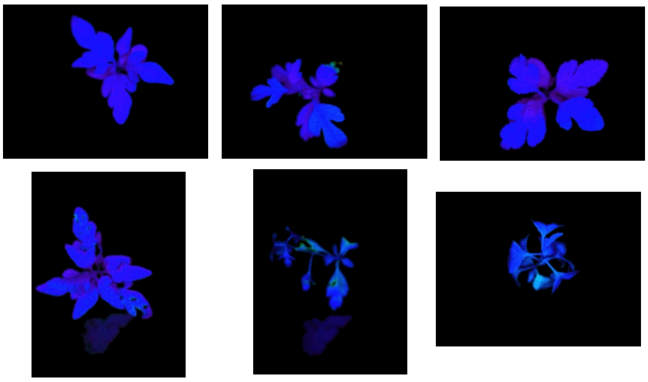

Fig.6d

Control p-TRV1 p-TRV2

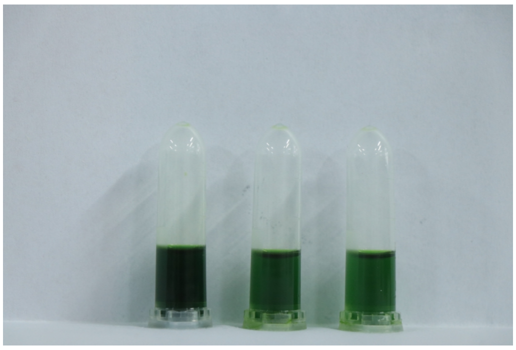

Fig.6h
